# ^18^F-FET-PET-guided gross total resection improves overall survival in patients with WHO grade III/IV glioma: moving towards a multimodal imaging-guided resection

**DOI:** 10.1007/s11060-021-03844-1

**Published:** 2021-10-01

**Authors:** Jonas Ort, Hussam Aldin Hamou, Julius M. Kernbach, Karlijn Hakvoort, Christian Blume, Philipp Lohmann, Norbert Galldiks, Dieter Henrik Heiland, Felix M. Mottaghy, Hans Clusmann, Georg Neuloh, Karl-Josef Langen, Daniel Delev

**Affiliations:** 1 Department of Neurosurgery, Medical Faculty, RWTH Aachen University, 52074 Aachen, Germany; 2 NAILA—Neurosurgical Artificial Intelligence Laboratory Aachen, Aachen, Germany; 3 Institute of Neuroscience and Medicine (INM-3, INM-4), Research Center Juelich, Juelich, Germany; 4 Department of Nuclear Medicine, Medical Faculty, RWTH Aachen University, 52074 Aachen, Germany; 5 Department of Neurology, Faculty of Medicine and University Hospital Cologne, University of Cologne, Cologne, Germany; 6 Department of Stereotaxy and Functional Neurosurgery, Faculty of Medicine and University Hospital Cologne, University of Cologne, Cologne, Germany; 7 Department of Neurosurgery, Medical Center, University of Freiburg, Freiburg, Germany; 8 Faculty of Medicine, Freiburg University, Freiburg, Germany; 9 JARA—Juelich Aachen Research Alliance, Juelich, Germany; 10 Center for Integrated Oncology Aachen Bonn Cologne Düsseldorf (CIO ABCD), Aachen, Germany

**Keywords:** Glioma, FET-PET, Extent of resection, Neurosurgery

## Abstract

**Purpose:**

PET using radiolabeled amino acid [^18^F]-fluoro-ethyl-_L_-tyrosine (FET-PET) is a well-established imaging modality for glioma diagnostics. The biological tumor volume (BTV) as depicted by FET-PET often differs in volume and location from tumor volume of contrast enhancement (CE) in MRI. Our aim was to investigate whether a gross total resection of BTVs defined as < 1 cm^3^ of residual BTV (PET GTR) correlates with better oncological outcome.

**Methods:**

We retrospectively analyzed imaging and survival data from patients with primary and recurrent WHO grade III or IV gliomas who underwent FET-PET before surgical resection. Tumor overlap between FET-PET and CE was evaluated. Completeness of FET-PET resection (PET GTR) was calculated after superimposition and semi-automated segmentation of pre-operative FET-PET and postoperative MRI imaging. Survival analysis was performed using the Kaplan–Meier method and the log-rank test.

**Results:**

From 30 included patients, PET GTR was achieved in 20 patients. Patients with PET GTR showed improved median OS with 19.3 compared to 13.7 months for patients with residual FET uptake (p = 0.007; HR 0.3; 95% CI 0.12–0.76). This finding remained as independent prognostic factor after performing multivariate analysis (HR 0.19, 95% CI 0.06–0.62, p = 0.006). Other survival influencing factors such as age, IDH-mutation, MGMT promotor status, and adjuvant treatment modalities were equally distributed between both groups.

**Conclusion:**

Our results suggest that PET GTR improves the OS in patients with WHO grade III or IV gliomas. A multimodal imaging approach including FET-PET for surgical planning in newly diagnosed and recurrent tumors may improve the oncological outcome in glioma patients.

**Supplementary Information:**

The online version contains supplementary material available at 10.1007/s11060-021-03844-1.

## Introduction

Gliomas are the most common primary tumors of the central nervous system after meningiomas, showing a prevalence rate of 47.6 per 100.000, and a worldwide annual incidence of 4–6 cases per 100,000 [1, 2], constantly increasing during the last two decades [3]. The WHO 2016 classification of tumors of the central nervous system classifies diffuse gliomas into WHO grade II and III astrocytoma, WHO grade II and III oligodendroglioma and grade IV glioblastoma [4]. Glioblastoma represents not only the most often occurring but also the deadliest malignant brain tumor, showing a mean overall survival (OS) of approximately 15 months despite maximal treatment [5–7]. The additional introduction of molecular markers like IDH mutation and 1p/19q co-deletion [8] has shown that the genetic background plays a pivotal role for oncological prognosis and treatment response, making them indispensable for tumor classification [4, 9, 10].

The treatment of patients with glioma of the WHO grades III or IV encompasses surgical resection, followed by radiotherapy and chemotherapy [11, 12]. Surgical resection plays a pivotal role in the glioma treatment by improving both, progression-free survival (PFS) and OS in those patients. Although a complete removal is practically impossible due to the gliomas’ infiltrative nature, an increasing amount of scientific work has shown a benefit in OS after gross total resection (GTR) [13–15]. GTR is defined by resection of contrast-enhancing (CE) tumor in early postoperative T1-weighted MRI images. However, gliomas grow beyond the CE borders, reducing the ability of MRI to determine the tumor burden. It has recently been shown that resection beyond gadolinium enhancement (supramarginal resection) results in longer OS and PFS (e.g., guided by 5-ALA) [16–18] raising the question whether GTR itself or the resection of biologically important tumor parts beyond CE contributes to the improved oncological course.

Molecular imaging using FET-PET can delineate the so-called biological tumor volume (BTV) [19, 20] and identify the most active (and most malignant) tumor parts [21, 22]. The potential of FET-PET to determine the extent of glioma resection has been investigated in several studies, and demonstrated additional information compared with conventional MRI [16, 23, 24]. A recent study in a larger series of patients with newly diagnosed glioblastoma demonstrated that preoperative BTV in FET-PET was larger than CE volume in MRI in 86% of the patients and in 10% of the patients increased FET uptake was present even outside areas of FLAIR hyperintensity [25]. Furthermore, it has been shown that a larger postoperative tumor volume in FET-PET is associated with poor prognosis in patients with glioblastoma [19]. This raises the question whether resection of BTV as depicted by FET-PET presents a meaningful target tissue to define GTR complementary to the area of CE in MRI [26–28]. Here, we hypothesize that extended removal of BTV defined as PET-GTR at any resection timepoint can positively influence the OS of patients with WHO grade III and IV gliomas. Therefore, using a semi-automated segmentation algorithm, we firstly looked for differences between tumor extension of preoperative BTV in FET-PET and CE MRI; and secondly investigated how complete or near-complete removal of BTV (= PET-GTR) defined as < 1 cm^3^ calculated residual BTV influences OS.

## Materials and methods

### Search criteria and description of patients’ characteristics

**Fig. 1:**
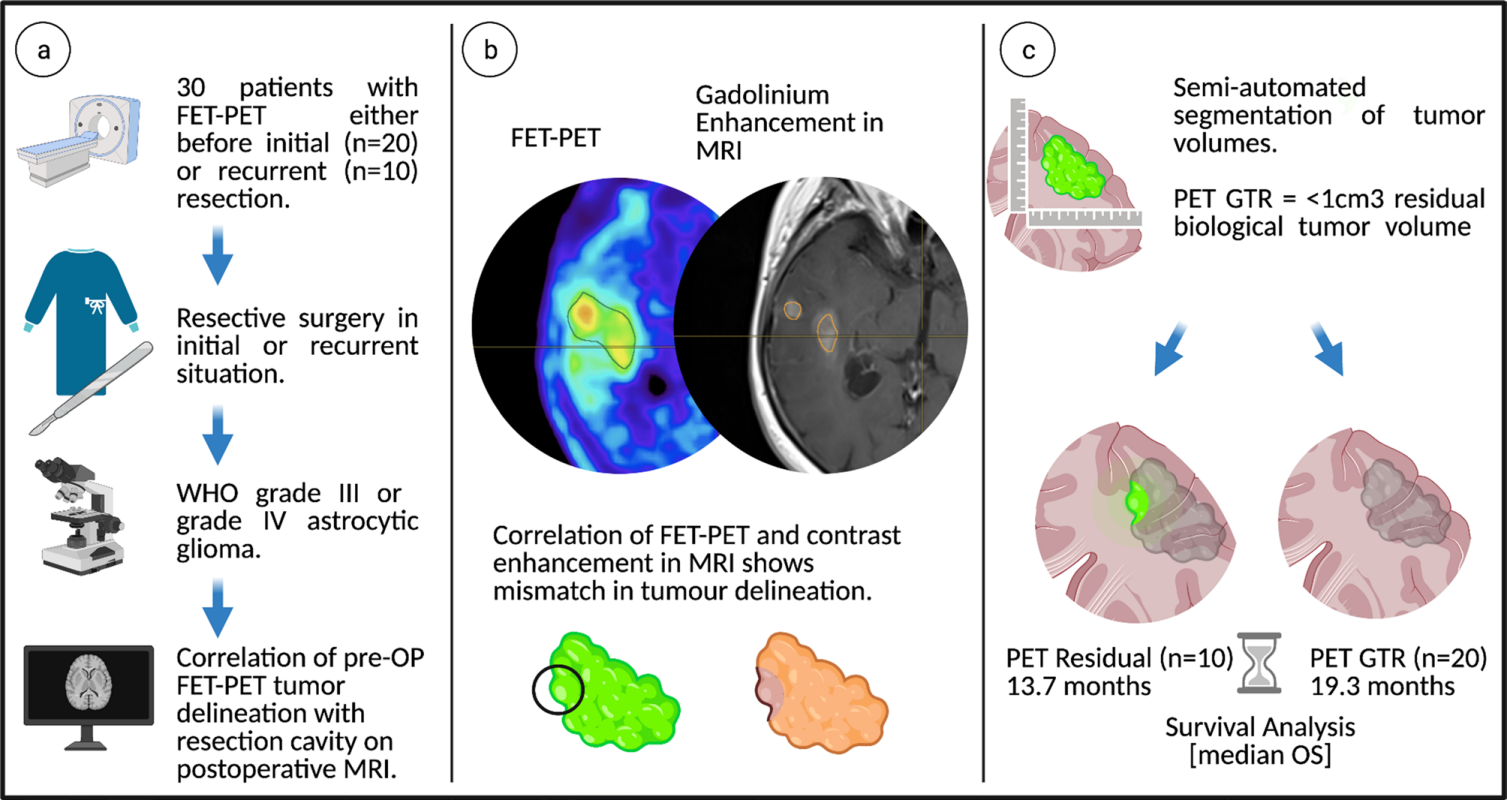
Patient cohort and study work-flow. **a** Patient identification with FET-PET examination either before initial or recurrent resection, histologically confirmed WHO grade III or IV glioma. **b** Comparison of FET-PET imaging with gadolinium T1-MRI shows mismatch between biological tumor volume and gadolinium uptake. **c** Calculated residual tumor volumes after segmentation and correlation with resection cavity. PET GTR is achieved with a residual biological tumor volume of < 1 cm^3^. Survival analysis between PET GTR and PET residual. Created with BioRender.com

Patients that received FET-PET at our center between 2015 and 2019 were screened for subsequent resective surgery of pathologically confirmed diagnosis of WHO grade III or IV glioma. 48 patients, operated between 2015 and 2019, were identified retrospectively, who underwent FET-PET examination before glioma surgery at our department. The mean time between preoperative FET-PET and surgery were 16.4 days (median 10.4 days). 30 patients received FET-PET before first tumor resection, and 18 patients underwent a FET-PET scan before surgery for tumor recurrence. MRI examinations were performed preoperatively with a mean of 14.3 days (median 13.5 days) before surgery. Postoperative MRI was obtained within 72 h after surgery, except in two cases where it was obtained within 96 h. We excluded patients with WHO grade II or oligodendroglioma (n = 11), patients who did not receive standard chemoradiation with temozolomide after initial resection (n = 2), patients without FET uptake (n = 1), and those with lacking information on follow-up, histopathological, or imaging data from the study (Fig. 1).

Thus, a cohort of 30 patients (20 with FET-PET before initial resection and 10 with FET-PET before recurrent resection) with WHO grade III or IV glioma was retrospectively identified.

### FET-PET acquisition and evaluation

As described previously, the amino acid FET was produced via nucleophilic ^18^F-fluorination with a radiochemical purity of greater than 98%, molar radioactivity greater than 200 GBq/µmol, and a radiochemical yield of about 60% [29]. According to international guidelines for brain tumor imaging using labeled amino acid analogues [30], patients fasted for at least 4 h before the PET measurements. All patients underwent a dynamic PET scan from 0 to 50 min post-injection of 3 MBq of FET per kg of body weight at baseline. PET imaging was performed either on an ECAT Exact HR + PET scanner (20 patients) in 3-dimensional mode (n = 64 scans; Siemens, Erlangen, Germany; axial field-of-view, 15.5 cm) or simultaneously with 3 T MR imaging using a BrainPET insert (10 patients, n = 15 scans; Siemens, Erlangen, Germany; axial field of view, 19.2 cm). The BrainPET is a compact cylinder that fits into the bore of the Magnetom Trio MR scanner [31, 32]. Iterative reconstruction parameters were 16 subsets, 6 iterations using the OSEM algorithm for the ECAT HR + PET scanner and two subsets, 32 iterations using the OP-OSEM algorithm for the BrainPET. Data were corrected for random, scattered coincidences, dead time, and motion, for both systems. Attenuation correction for the ECAT HR + PET scan was based on a transmission scan, and for the BrainPET scan on a template-based approach [33]. The reconstructed dynamic data sets consisted of 16-time frames (5 × 1 min; 5 × 3 min; 6 × 5 min) for both scanners.

To optimize the comparability of the results related to the influence of the two different PET scanners, reconstruction parameters, and post-processing steps, a 2.5 mm 3D Gaussian filter was applied to the BrainPET data before further processing. In phantom experiments using spheres of different sizes to simulate lesions, this filter kernel demonstrated the best comparability between PET data obtained from the ECAT HR + PET and the BrainPET scanner [34].

### Quantification of tumor volumes

For the evaluation of FET-PET data, summed PET images over 20–40 min post-injection were used. A crescent-shaped reference ROI, placed in the contralateral hemisphere in an area of normal-appearing brain tissue served as background region. The BTV was determined by a three-dimensional auto-contouring process using a threshold of 1.6 above the reference value. This has been described to best separate primary tumor from non-tumoral tissue in a biopsy-controlled study [20].

For image analysis and acquisition of tumor volumes, we used a commercial imaging software with a half-automated segmentation tool (“Brainlab Elements” including the “smart brush” module, Brainlab AG, Munich, Germany [35]) as it is commonly done for surgical planning. CE tumor volume (pre- and postoperatively) and the preoperative BTV were segmented by two independent observers: Preoperative MRI and FET-PET images were co-registered to determine differences in tumor delineation by both modalities. Preoperative FET-PET and postoperative MRI images were co-registered to determine residual FET-PET tumor parts. For this we visually compared pre-operative segmented FET-PET tumor volume with postoperative resection cavity volume. If the preoperative FET-PET tumor volume was not completely localized within the resection cavity, we designated it as residual BTV (i.e., PET residual). Next, we obtained volumetries of the residual BTV. All co-registration procedures were performed using a commercial fusion algorithm (“Brainlab Elements Image Fusion”, Brainlab AG, Munich, Germany [36]). No additional correction for post-operative brain-shift was performed. To balance for inaccuracies of the semi-automated segmentation and limitations of FET-PET resolution, we defined threshold of less than 1 cm^3^ residual BTV as *FET-PET GTR* (*PET GTR*) and a residual BTV of at least 1 cm^3^ as *PET residual*. For CE volume, a resection of more than 90% of the pre-surgical volume was considered as gross-total [37, 38].

### Statistical analysis

For statistical analysis, we used the open-source statistical software Jamovi (version 1.2) and Python including the libraries Numpy and Lifelines. In any given analysis, a p-value of < 0.05 was considered statistically significant. For comparison of patient characteristics, Fisher’s exact test was used for categorical data and the Mann–Whitney-U test for continuous data respectively. Survival analysis was performed using Kaplan–Meier-Curves and the log-rank test. We used a Cox proportional hazards regression model for multivariable survival analysis.

## Results

### Cohort description

30 patients (11 females) with WHO grade III or IV gliomas with a median age of 59.0 years (interquartile range: 53.0–63.0) were included in the study. All patients underwent surgical resection followed by a sequential or combined radio-chemotherapy. At time of analysis, 25 patients had died and 5 patients where censored (the mean follow-up time for censored patients was 21.65 months).

### Biological tumor volume defined by FET-PET corresponds only partially to the CE delineation of the tumor

In one case only, FET-PET was completely within the tumor area as defined by CE. In 19 cases both modalities partially overlapped and in 10 cases FET-PET was exceeding CE delineated tumor borders entirely. Only one case did not show any CE.

PET GTR was achieved in 20 patients, whereas in the remaining 10 patients the BTV was incompletely resected (PET residual). Both groups differed in the pre-surgical extent of BTV as depicted by FET-PET with a median of 9.2 cm^3^ (interquartile range: 7.5–11.0) for patients with PET GTR compared to 26.3 cm^3^ (interquartile range: 12.9–38.3) for patients with PET residual (Mann–Whitney-U test, p-value = 0.011) (*Table **1**)*. Further, both groups differed in the number of mid-line crossing tumors (5% in PET GTR vs 30% in PET residual, Fischer’s exact test p-value = 0.095) and eloquent located tumors (65% PET GTR vs 90% PET residual, Fishers’ exact text p-value = 0.210), albeit the differences were not statistically significant.

**Table 1:** Cohort characteristics

	Total	PET GTR (< 1 cm^3^ residual BTV)	PET residual (≥ 1 cm^3^ residual BTV)	p-value
n	30	20	10	
Age at diagnosis (years median)	59.0 [53.0–63.0]	59.0 [53.0–63.0]	60.0 [49.8–66.0]	.758^#^
Male/Female (%)	19/11 (63.3/36.7)	13/7 (65.0/35.0)	6/4 (60.0/40.0)	1.000^§^
WHO grade III/IV	5/25	3/17	2/8	1.000^§^
MGMT methylated (%)	17 (58.6), n = 29*	12 (60.0)	5 (55.6), n = 9*	1.000^§^
IDH mutated (%)	5 (16.7)	3 (15.0)	2 (20.0)	1.000^§^
pre-OP BTV (cm^3^, median)	10.8 [8.5–25.7]	9.2 [7.5–11.0]	26.3 [12.9–38.3]	**.011**^#^
pre-OP CE volume (cm^3^, median)	7.44 [3.1–25.2]	6.44 [3.4–13.4]	24.7 [3.7–39.0]	.143^#^
CE rest (cm^3^, median)	0.1 [0.0–1.3]	0.0 [0.0–0.7]	0.8 [0.1–1.6]	.150^#^
Initial/recurrent resection (%)	20/10 (66.7/33.3)	12/8 (60.0/40.0)	8/2 (80.0/20.0)	.419^§^
Mid-line crossing (%)	4 (13.3)	1 (5.0)	3 (30.0)	.095^§^
Hemisphere left/right (%)	20/10 (66.7/33.3)	13/7 (65.0/35.0)	7/3 (70.0/30.0)	1.000^§^
Eloquent localization (%)	22 (73.3)	13 (65.0)	9 (90.0)	.210^§^

Both PET-GTR and PET residual groups were tested for significant differences (bold). Continuous data is represented as median with interquartile range in []

*For one patient no MGMT methylation status was available

^#^Mann–Whitney-U. ^§^Fisher’s exact test

### GTR of FET-PET tumor volume is associated with longer overall survival (OS)

**Fig. 2:**
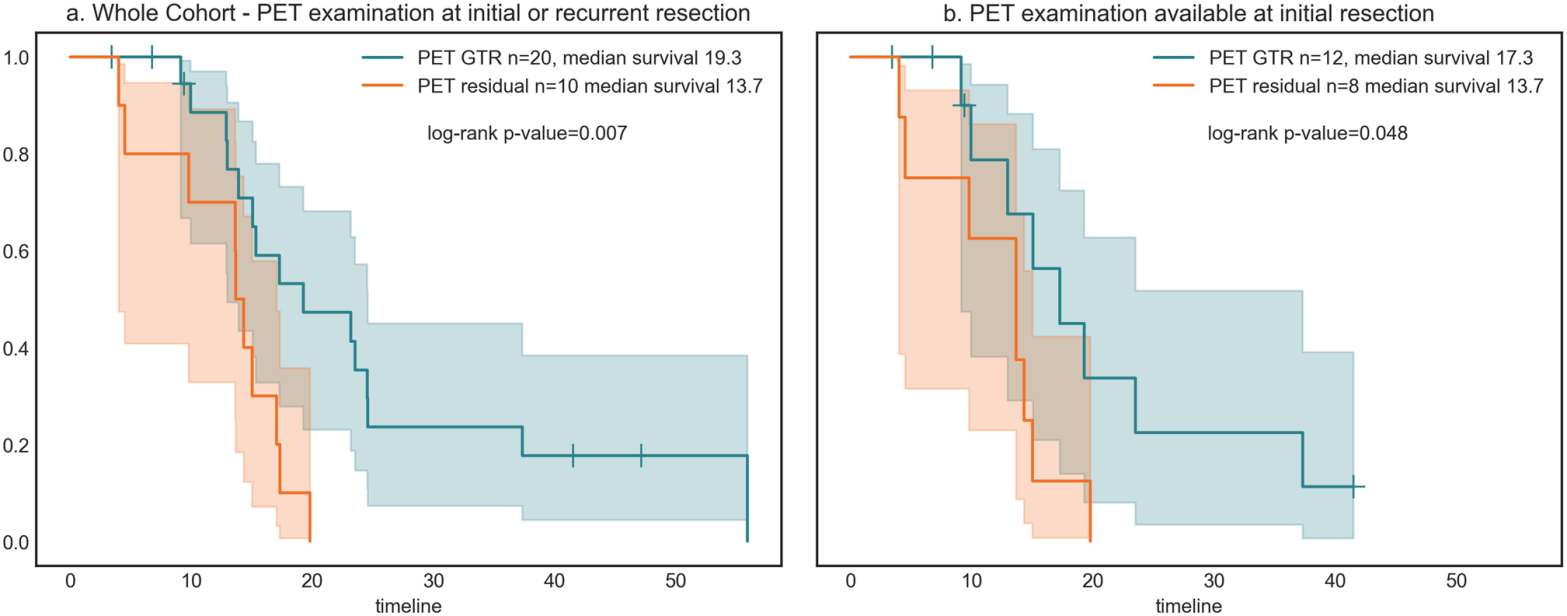
Kaplan–Meier curves of the whole cohort. **a** PET GTR results in longer overall survival (19.3 months) compared to patients with PET residual (13.7 months), p-value = 0.007. **b** Patients with FET-PET examination before initial resection, showing a significant effect of PET GTR

Aiming to answer the question whether complete resection of BTV in newly diagnosed and recurrent gliomas would influence patients’ OS as calculated from the time point of initial diagnosis, we performed survival analysis over the whole cohort. Patients with PET GTR showed longer OS with a median survival of 19.3 months compared to patients with PET residual with a median survival of 13.7 months (p = 0.007; HR 0.3 [0.12–0.76]) (Fig. 2a). Both groups showed no statistically significant differences in age at primary diagnosis, WHO-grading, histopathology, MGMT methylation status, IDH mutation frequency, pre-operative contrast-enhancing tumor volume (CE volume), or residual contrast-enhancing tumor volume (CE residual) (Table 1). However, pre-operative CE volume notably differed in both groups although this finding was not statistically significant.

FET-PET was performed in 20 patients before initial resection of newly diagnosed gliomas and in 10 patients before resection of tumor recurrence. To exclude the influence of the timepoint at which PET GTR was performed, we approached the cohort in three different ways.

Firstly, we calculated the median OSs within the two groups (*before initial resection* vs. *before recurrent resection*) showing that the survival benefit of PET GTR remained independent of the examination timepoint. In the subgroup of patients with FET-PET prior to initial resection, OS for PET GTR was 17.3 months compared to 13.7 months for PET residual. In the subgroup of patients with FET-PET prior to recurrent resection OS for PET GTR was 23.9 months compared to 17.2 months for PET residual.

Secondly, we performed a survival analysis within the subgroup of patients with FET-PET prior to initial resection (Fig. 2b), showing a survival benefit (p = 0.048; HR 0.35 [0.12–1.04]) for patients with PET GTR. Due to the small patient number (n = 10) a survival analysis in the subgroup of patients with FET-PET prior to recurrent resection was not possible.

Further, we investigated for the subgroup of WHO grade IV tumors (n = 25). For this subgroup PET GTR (n = 17) showed a median survival of 17.3 months vs. 13.7 months for the PET residual group (n = 8) (p = 0.005; univariable HR 0.25 [0.09–0.71]) (Supplementary Fig. 1a). Patients with a WHO grade IV tumor and available PET examination at initial diagnosis (n = 16) also showed improved survival for PET GTR (n = 9) with 15.1 months median OS compared to 13.6 months for PET residual (n = 7) (p = 0.048, univariable HR 0.27 [0.07–1.08]) (Supplementary Fig. 1b). For WHO grade IV patients with available PET examination before recurrent resection (n = 9) only one individual was identified as PET residual, thus excluding reasonable comparative survival analysis.

Finally, we included the timepoint of FET-PET examination as variable in a multivariate analysis, which showed that FET-PET timepoint has no significant effect as independent variable on the OS (Fig. 4).

**Fig. 3:**
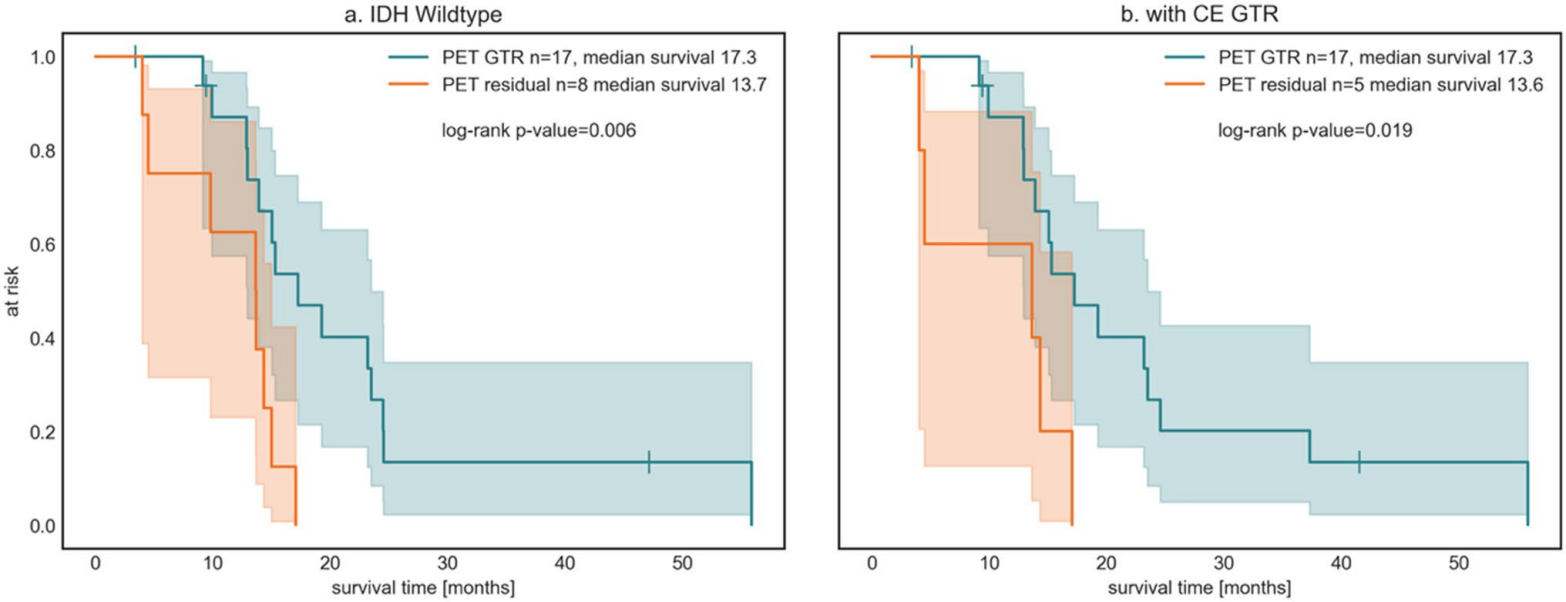
Subgroup analysis. **a** Patients with IDH-wildtype that received PET GTR showed improved OS (17.3 months) compared to patients with PET residual (13.7 months). **b** The same effect could be observed in the subgroup of patients that received GTR as defined by gadolinium uptake (PET GTR: 17.3 months OS vs. PET residual: 13.6 months OS)

A further subgroup analysis of patients with IDH-wildtype glioma revealed this effect to be consistent. PET GTR (n = 17) showed a median survival of 17.3 months vs. patients with PET residual (n = 8) with 13.7 months (p = 0.006; univariable HR 0.25 [0.09–0.72]) (Fig. 3a).

Aiming to investigate the additional value of FET-PET extent of resection (EOR) we stratified the cohort only for those patients, who underwent complete resection of the gadolinium contrast enhancing tumor parts (CE GTR, n = 22). In this subgroup of patients with CE GTR, the additional PET GTR resulted in a median OS of 17.3 months compared to 13.6 months in patients with PET residual (p = 0.019, univariable HR 0.27 [0.08–0.87]) (Fig. 3b).

### PET GTR is an independent prognostic factor associated with longer overall survival in multivariate regression model

**Fig. 4:**
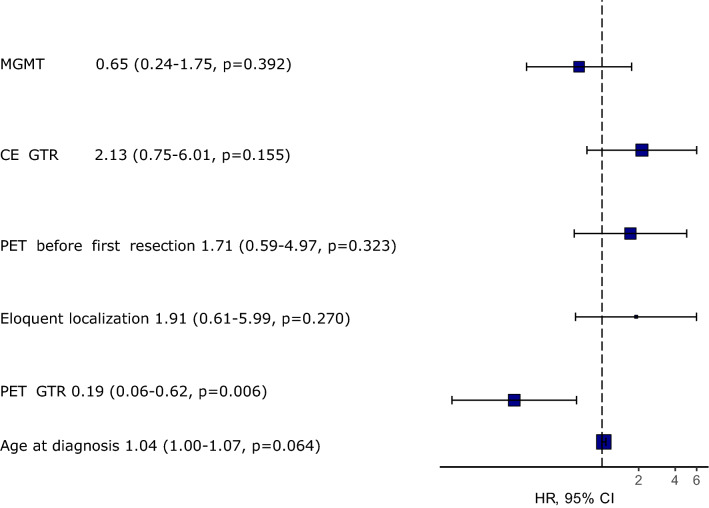
Hazards regression plot of multivariable survival analysis. PET GTR is the only prognostic factor identified to reduce hazard (p = 0.006, Cox proportional hazards regression model)

Multivariable survival analysis using a Cox proportional hazards regression model was performed for the whole cohort with prognostic factors known to influence the OS: MGMT promotor methylation status, timepoint of FET-PET examination, PET GTR, CE GTR and age. Only PET GTR remained as independent prognostic factor associated with improved OS (HR = 0.19 [0.06–0.62], p = 0.006) (Fig. 4). Multivariate analysis was performed using different models including pre-operative BTV, pre-operative CE volume, PET GTR, CE GTR and IDH mutation. Only IDH mutation as a well-established prognosis parameter showed a reduced HR of 0.23 ([0.06–0.86], p = 0.03). Sex did not show any significant difference as risk factor in multivariate analysis.

## Discussion

Surgical resection plays a pivotal role in the treatment of glioma since the EOR correlates with patients’ OS. While conventional CE-MRI delineates the tumor volume of glioma mainly according to the distorted blood–brain barrier, molecular imaging techniques can improve tumor delineation by detecting the biologically active tumor parts. PET using radiolabeled amino acids such as FET-PET has been shown to provide an excellent delineation of the BTV, which may substantially differ in both, tumor localization and extension, compared to MRI [25, 39, 40]. Here we present data of patients, who have undergone a FET-PET examination before surgical resection of WHO grade III and IV astrocytic glioma and show that (1) the extent of FET-PET differs from CE MRI and (2) PET GTR resection influences OS of those patients.

### PET GTR results in improved OS

In our study, GTR of FET-PET positive tumor tissue resulted in significantly improved OS. While the predominant indications for the use of FET-PET in higher-grade glioma are differentiation between tumor progression and pseudo-progression, tumor extent delineation, and precise planning of biopsies [41], our results support the view that amino acid can be particularly useful for the initial surgical resection planning. Similar results were shown by Pirotte et al. in 2009 in a group of 66 glioma patients that showed significant longer OS after complete removal of ^18^F-fluorodeoxyglucose- (FDG) or ^11^C-methionine-PET-positive volumes [39]. Regarding FET-PET, Suchorska and colleagues showed in the context of radio-chemotherapy that pre-therapeutical BTV is a strong prognostic factor for PFS and OS, and, most recently, Muther et al. reported that a calculated postoperative residual BTV of < 4.3 cm^3^ resulted in significant better PFS and OS after fluorescence-guided resection in GBM patients [16, 19].

PET GTR remained as independent prognostic factor associated with better OS after correcting for multivariate analysis. The effect of PET GTR was even stronger than the one of CE GTR arguing for the importance of resecting in particular active tumor tissue, which might be missed by CE alone [26]. Although both groups notably differed in preoperative CE tumor volume, postsurgical CE volumes were only marginally larger in the PET residual group. This suggests comparably good resectability of CE delineated tumor in both groups.

The only statistically significant difference in both groups was the preoperative BTV. Since our analysis is based on the residual tumor volumes this difference does not influence our results.

### Multimodal image-guided resection as prerequisite for personalized treatment

Recent molecular data have shown that malignant tumors are defined by their heterogeneity and the better visualization and understanding of this heterogeneity may uncover more personalized treatment regimens [4, 9]. The evident differences in tumor volume and spatial dissemination as delineated by CE MRI vs. FET-PET strongly advocate an expansion of clinical usage of both modalities [25, 40, 42]. A multimodal approach potentially exploiting information including gadolinium MRI, FET-PET, and potentially functional parameters might augment our standard definition of ‘the tumor’ and help to tailor the surgical plan and translate it to maximal safe GTR and better oncological outcome [22, 43]. Therefore, recent advances in neuro-oncological imaging, as well as increasing integration of machine and deep learning methods, support the use of multimodal approach, in particular with a focus on molecular imaging [44–50].

## Limitations

The retrospective nature and small patient number are significant limitations of the current work. However, the effect of PET GTR remained stable in both, subgroup and multivariate analysis, implying its importance. Both groups showed some differences in pre-operative CE, and eloquent locations of the tumors. Although these findings were not statistically significant, this poses a potential bias that needs to be acknowledged in the evaluation of this data. The heterogeneity of our cohort also limits the generalizability of our results. Another constraint is the only limited information regarding molecular markers beyond IDH and MGMT. The co-registration of pre-OP FET-PET volumes with the post-OP MRI scans may lead to methodological inaccuracies induced by brain-shift and image-fusion inaccuracies, e.g., small areas of FET-PET tumor delineation sometimes only slightly exceed the resection cavity. Since we expected an improved clinical outcome to result from a small absolute residual FET-PET volume post-OP, we tried to correct for these by introducing 1 cm^3^ “margin of error” during the evaluation of the images. A similar approach has already been used by others as well [16]. The most accurate way to evaluate post-OP residual BTV would be postoperative FET-PET imaging. This lack of postoperative PET images is certainly an important limitation, however, there is still a significant scientific uncertainty at which time after surgery FET-PET shows residual tumor rather than reactive postoperative changes [24, 51, 52]. Furthermore, a recent study reported on a postoperative flare phenomenon with increased FET uptake the cause of which is not yet clear [23].

## Conclusion

PET GTR improves OS in WHO grade III and IV gliomas by removing additional active tumor areas beyond CE MRI borders. Therefore, a multimodal imaging approach consisting of MRI and FET-PET can help to achieve a better tumor delineation and consequently better tumor resection, thus positively influencing the oncological outcome. To further improve the evidence for a multimodal imaging approach for surgical planning including FET-PET, a prospective study is needed.

## Supplementary Information

Below is the link to the electronic supplementary material.

Supplementary file1 (DOCX 130 kb)

## Data Availability

The datasets generated during and/or analysed during the current study are available from the corresponding author on reasonable request.

## Code availability

Custom code used in the analysis of the current study are available from the corresponding author on reasonable request.
