# Supplementary material for: 18F-FET-PET-guided gross total resection improves overall survival in patients with WHO grade III/IV glioma: moving towards a multimodal imaging-guided resection
Source: J Neurooncol. 2021 Oct 1;155(1):71–80. doi: 10.1007/s11060-021-03844-1 (PMC8545732; doi:10.1007/s11060-021-03844-1)
Supplement: Supplementary file 1 — Supplementary file1 (DOCX 130 kb) [file 11060_2021_3844_MOESM1_ESM.docx]

**Supplementary Figure 1**

**
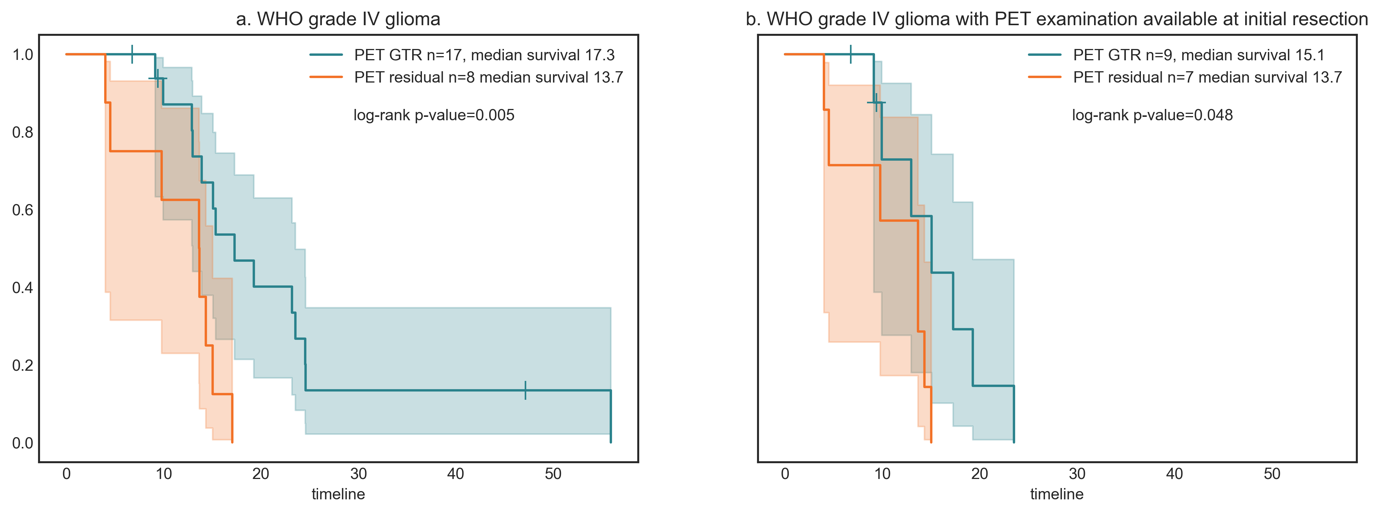
**

**Suppl. Fig 1.** *Analysis of WHO grade IV glioma subgroups. A. In the subgroup of WHO grade IV glioma (n=25) PET GTR resulted in significantly improved OS. B. This observation was confirmed for patients observed at initial resection only (n=16).*
